# Cardiac Depression Scale: Mokken scaling in heart failure patients

**DOI:** 10.1186/1477-7525-10-141

**Published:** 2012-11-23

**Authors:** Chantal F Ski, David R Thompson, David L Hare, Andrew G Stewart, Roger Watson

**Affiliations:** 1 Cardiovascular Research Centre, Australian Catholic University, Melbourne, Australia; 2 Department of Medicine, University of Melbourne and Department of Cardiology, Austin Hospital, Melbourne, Australia; 3 School of Nursing, University of Hull, Hull, United Kingdom

**Keywords:** Cardiac Depression Scale, Heart failure, Depression, Mokken scaling

## Abstract

**Background:**

There is a high prevalence of depression in patients with heart failure (HF) that is associated with worsening prognosis. The value of using a reliable and valid instrument to measure depression in this population is therefore essential. We validated the Cardiac Depression Scale (CDS) in heart failure patients using a model of ordinal unidimensional measurement known as Mokken scaling.

**Findings:**

We administered in face-to-face interviews the CDS to 603 patients with HF. Data were analysed using Mokken scale analysis. Items of the CDS formed a statistically significant unidimensional Mokken scale of low strength (H<0.40) and high reliability (Rho>0.8).

**Conclusions:**

The CDS has a hierarchy of items which can be interpreted in terms of the increasingly serious effects of depression occurring as a result of HF. Identifying an appropriate instrument to measure depression in patients with HF allows for early identification and better medical management.

## Background

Heart failure (HF) is a leading cause of morbidity and mortality worldwide that imposes a considerable human and economic burden [1-4]. Depression and depressive symptoms are common in patients with HF though estimates of prevalence vary depending on method and timing of assessment [5,6]. A meta-analytic review on depression in HF reported clinically significant depression in 21.5% of patients, but varied by use of questionnaires versus diagnostic interview and HF severity [6]. Accurate identification of depression in patients with HF is critical because of significant associations with increased hospitalisations [5,7,8], poorer functional limitations [9,10], lower survival rates [8,11,12], and reduced quality of life [13]. Despite an American Heart Association (AHA) advisory recommending depression screening for all patients with coronary heart disease [14], depression remains under recognised and under treated in this population [15]. Given the potential health consequences of untreated comorbid HF and depression, the value of using reliable and valid instruments to measure depression cannot be underestimated. This is paramount when one considers the numerous indirect costs of depression such as absenteeism, decreased productivity, increased risk for secondary complications, and malignant effects on dependent family members [16,17].

Recognition of depression in patients with HF is complicated by an overlap in risk factors such as smoking, excessive alcohol consumption, obesity, and lower physical activity, and symptoms such as fatigue, lack of interest in activities, appetite gain or loss, psychomotor impairment, poor concentration, and depressed mood [1,18]. These similarities have the potential to result in contrasting outcomes such as screening for depression generating a number of false positive results, and depression remaining undiagnosed for a substantial period of time. Hence, accurate assessment of depression in patients with HF is necessary to aid early intervention.

The AHA advisory endorsing depression screening, referral, and treatment of all individuals with coronary heart disease [14] has provoked considerable debate [19-23], with much of the contention surrounding the lack of evidence regarding the choice of instruments to measure depression, specifically their psychometric properties. Of note, is that depression screening will only identify depressive symptoms, i.e. those at risk of depression, as a diagnosis of depressive illness can only be confirmed when a person has a number of depressive symptoms consistently over a couple of weeks or more.

The Cardiac Depression Scale (CDS) was developed for the specific purpose of providing a valid and reliable instrument to measure depression in cardiac patients [24]. The CDS is the only measure for depression derived from responses of cardiac patients. It offers a more responsive and sensitive alternative to other measures that are not always suitable for cardiac patients because they have been developed and validated in non-cardiac populations [25]. It is an easily administered (taking five minutes to complete and one minute to score) disease-specific, self-rating scale that assesses the full range of depression seen in cardiac patients. Whilst having 97% sensitivity and 85% specificity for diagnosing major depression, it also assesses the range of less severe ‘reactive’ depression [26]. Validation studies have applied the CDS to a general cardiac population and encouraged its continued use and evaluation in other cardiac patient groups [27-29]. Thus, we aimed to validate the CDS in patients with HF using Mokken scaling analysis (MSA), a method related to item response theory that analyses multivariate databases for unidimensional hierarchies of items [30].

A unidimensional hierarchy of items can be identified using Loevinger’s coefficient (H) along with other indicators of reliability, probability and monotone homogeneity of items [31]. Recent versions of software for MSA are capable of analysing for invariant item ordering (IIO) [32], an important property of questionnaires whereby, for a set of items displaying IIO, every respondent responds to all of those items in exactly the same order in the hierarchy [33]. IIO is not necessary for the utility of a questionnaire showing a Mokken scale of items: unidimensionality and monotone homogeneity are sufficient as such a scale can be used to order individuals. However, IIO is desirable. Mokken scaling has recently been applied to several scales designed to measure psychological morbidity and other phenomena [34-36]. We investigated if a hierarchy of items, according to Mokken scaling criteria, existed in the CDS when applied to patients with HF. This paper contributes to a series of validation papers [34-38] that have implemented MSA to determine scale item unidimensional hierarchy.

## Methods

We administered the CDS in face-to-face interviews to 603 HF patients attending the out-patient heart failure clinic of a major metropolitan hospital. Gender (male 68% and female 32%) and age distributions (*x* = 70, SD = 14) were typical for this patient group. As part of the usual clinic assessment protocol developed by one of the authors, all patients had the CDS routinely administered by a cardiac nurse prior to seeing the heart failure cardiologist. Clinical characteristics of study participants are outlined in Table 1.

**Table 1:** Summary characteristics of heart failure patients (n=603)

	**n**	**%**
**DIAGNOSIS**		
HFrEF	476	79
HFpEF	49	8
Valvular & RHF	16	3
Other	62	10
**AETIOLOGY** (HFrEF)		
Ischemic	259	54
Non-ischemic	167	35
Unknown	50	11
**NYHA CLASS**		
I	114	19
II	247	41
III	102	17
IV	11	2
Unknown	129	21

HFrEF = heart failure with reduced ejection fraction.

HFpEF = heart failure with preserved ejection fraction.

RHF = right heart failure.

The study was approved by the institutional Human Research Ethics Committee (Approval No. H2006/02657). Written informed consent was obtained from the participants.

### CDS

The CDS is a 26 item questionnaire, each item requiring a response on a Likert scale from 1 (‘Strongly disagree’) to 7 (‘Strongly agree’), the scale being tagged at each end with an explanatory phrase. Respondents answered questions, in an interview, and indicated the appropriate number on the Likert scale which expresses the strength of their response to the item. To avoid a fixed response set, some items are positively worded (e.g. ‘My concentration is as good as it ever was’) and others negatively (e.g. ‘I may not recover completely’). The seven positively worded items were reverse coded for aggregation and statistical analysis.

### Mokken scaling

Data were analysed using the commercially available software Mokken Scaling Analysis for Polytomous items (MSP) for Windows version 5.0 [39] and the MSA feature in the public domain software R [40]. Data were converted from SPSS into the formats required for each of these programs and the MSP was used to identify unidimensional hierarchies of items which were subsequently analysed of IIO using the MSA in R (the statistical programme R). Using MSP the data were run using Loevinger’s coefficients from H=0.05 in 0.05 increments to H=0.45 to test for multidimensionality [41]; only one dimension was identified and this was subsequently run using the default settings of p<0.05 and H=0.30.

## Results

The results of the MSA are shown in Table 2; 22 items formed a unidmensional Mokken scale of low strength (H<0.40) but high reliability (Rho>0.8) which was statistically significant. Fifteen items showed IIO but at low30 accuracy (H^T^<0.). The hierarchical pattern of responses to items in the Mokken scale, ordered by their mean scores in terms of difficulty from low difficulty (high mean score) to high difficulty (low mean score) is interpretable in terms of respondents more easily endorsing items related to general concern about their condition (for example: ‘My problems are not over yet’; ‘I am not the person I used to be’; ‘I may not recover completely’) through a range of items to serious concerns about their condition with a sense of hopelessness (for example: ‘Things which I regret about my life are bothering me’; ‘There is only misery in the future for me’) and even expressions of wishing for death (‘Dying is the best solution for me’).

**Table 2:** Mokken scaling of the Cardiac Depression Scale (n=603)

**Item**	**Label**	**Mean**	**H**
11	Dying is the best solution for me	2.07	0.31†
14	There is only misery in the future for me	2.48	0.43†
18	Things which I regret about my life are bothering me	2.78	0.30†
12	I feel in good spirits*	3.00	0.39†
23	I feel independent and in control of my life*	3.13	0.35†
4	I get pleasure from life at present*	3.24	0.35†
20	My memory is as good as it always was*	3.25	0.31†
2	My concentration is as good as it ever was*	3.30	0.35†
15	My mind is as fast and alert as always*	3.30	0.32†
19	I gain as much pleasure from my leisure activities as I used to*	3.49	0.38†
10	I feel like I’m living on borrowed time	3.52	0.40†
24	I lose my temper more easily nowadays	3.52	0.31†
16	I get hardly anything done	3.64	0.40
3	I can’t be bothered doing anything much	3.65	0.34†
1	I have dropped many of my interests and activities	3.67	0.35†
22	I seem to get more easily irritated by others than before	3.88	0.37†
25	I feel frustrated	3.89	0.42
6	I may not recover completely	3.90	0.33
8	I am not the person I used to be	4.17	0.42
17	My problems are not over yet	4.39	0.36

H = 0.36; Rho = 0.92; p= 0.000082; H^T^=0.18 for items (**†**) showing invariant item ordering; NB asterisked items (*) are reverse scored.

## Discussion

Depression in patients with HF is associated with increased hospitalisations, decreased medication adherence, poorer health outcomes, increased mortality, and significant economic costs [5-13]. The high prevalence of depression in patients with HF [6] and the AHA advisory to screen for depression [14] indicates the need for a valid and reliable instrument. We provide evidence that the CDS is an appropriate prognostic indicator for identifying depression in patients with HF.

Although most parameters in biological systems are continuous variables, for pragmatic purposes we also heuristically organise information into identifiable groups. Thus, whilst symptoms of depressed mood and the components of diagnosed “depression’ are actually continuous variables, the diagnosis of “depression” has a dichotomous meaning that is useful for both management and determining prognosis. Whilst the CDS was originally developed to measure the full range of depressive symptoms in cardiac patients, it also has excellent accuracy for the “diagnosis” of major depression as a clinical entity (26). Nevertheless this current study was designed to examine the utility of the CDS over the full range of depressive symptoms, with the finding of a gradation from easily endorsed items through to those items representing more severe depression.

The application of Mokken scaling to the CDS has demonstrated the existence of a hierarchy of items which can be interpreted in terms of the increasingly serious effects of depression occurring as a result of a HF. Unresolved problems, frustration and irritability are at one end of the hierarchy, whilst guilt, misery and wanting to die (measures of severe depression) are at the other. The demonstration of this hierarchy provides new information (a non-parametric measure of IRT) about the structure of the CDS and adds utility to the scale in clinical practice when managing patients with HF.

Establishing unidimensional sets of items *per se* in assessment instruments is useful in demonstrating underlying dimensions and determining which items are related to those dimensions. The CDS, being derived from the responses of cardiac patients themselves, provides a distinctive base from which to assess item hierarchy based on the level of difficulty of items for patients with HF. However, hierarchies of items add further value: they are inherently useful because scores calculated from such sets of items help to discriminate between people better than scores from sets of items where no such hierarchy exists. A score on a hierarchical set of items indicates better the level of the latent trait because a score is related to a specific set of items, thereby providing descriptors to the level of the latent trait present. Without demonstrating that such hierarchies exist—and some sets of items are resistant to this—it is impossible to tell, for example, if two people with the same score on the instrument are both at the same level on the latent trait. If the items are not hierarchical then any set of items could contribute to any particular score and in instruments such as the CDS, these items will represent different levels of severity.

Invariant item ordering was not demonstrated for the items retained in the Mokken scale of the CDS; this means that there is no guarantee that all (HF) respondents to the CDS will respond to the hierarchy of items in precisely the same order. This could be partly explained as, within each patient cohort, there are patients who have pre-existing chronic depression as well as those with a more acute 'adjustment disorder" who might respond more strongly to a slightly different range of items. Also the lack of IIO indicates that the conceptual distance between the items is likely to be small and that there is some overlap of the item response functions because of cohesion in the scale items. Nevertheless, the Mokken scale is still useful for ordering respondents on the basis of their mean scores.

## Conclusions

In conclusion, we found the CDS, developed as a valid and reliable cardiac-specific measure for depression, to contain a set of items that when applied to patients with HF are hierarchical. The CDS is an appropriate depression screening instrument for patients with HF that allows for early identification and better management, especially of those at increased risk of functional limitations, mortality, and impaired quality of life.

## Abbreviations

CDS: Cardiac Depression Scale; HF: Heart failure; AHA: American Heart Association; MSA: Mokken scaling analysis; MSP: Mokken scaling analysis for polytomous items; H: Loevinger’s coefficient; IIO: Invariant item ordering; SPSS: Statistical Package for the Social Sciences.

## Competing interests

The authors declare that they have no competing interests.

## Authors’ contributions

CFS participated in the study design and drafted the manuscript. DRT conceived and coordinated the study, participated in its design and helped to draft the manuscript. AGS participated in the study design and collected the data. DLH participated in the design of the study and helped to draft the manuscript. RW conducted the Mokken scaling analysis and helped to draft the manuscript. All authors read and approved the final manuscript.
